# The Role of Videogame Micro-Transactions in the Relationship Between Motivations, Problem Gaming, and Problem Gambling

**DOI:** 10.1007/s10899-024-10365-9

**Published:** 2024-11-13

**Authors:** Erin Gibson, Mark D. Griffiths, Filipa Calado, Andrew Harris

**Affiliations:** International Gaming Research Unit, Psychology Department, Nottingham Trent University, 50 Shakespeare Street, Nottingham, NG1 4FQ UK

**Keywords:** Micro-transactions, Videogames, Problem gambling, Gaming disorder, Self-determination, Motivations

## Abstract

Emerging research has highlighted potential associations between micro-transaction use and problematic videogame and gambling behaviour. An increasingly prominent theory highlights that self-determined motivations and basic psychological needs may play crucial roles in the development of problematic videogame and gambling behaviour. However, literature discussing the role that micro-transaction use has in this relationship is scarce. The present study examined the role of micro-transactions in the relationship between self-determined motivations for gaming and gambling and problematic behaviour (internet gaming disorder and problem gambling severity). A sample of 370 participants (74.1% male, *M*_age_ = 28.24 years, *SD* = 7.88) answered questions related to their gaming and gambling motivations, basic psychological needs, micro-transaction use (i.e., type of micro-transaction, expenditure, and frequency of use), internet gaming disorder, and problem gambling. The present study used structural equation modelling methods to test relationships between these variables. The results indicated positive associations between extrinsic gaming and gambling motivations and frequency of micro-transaction use. Frequency of micro-transaction use (i) partially mediated the relationship between extrinsic gambling motivations and problem gambling severity, and (ii) fully mediated the relationship between externally regulated gaming motivations and problem gambling severity. Expenditure on micro-transactions and basic psychological needs were not found to be significant variables in the present study. Potential explanations for the findings, including a lack of self-esteem and a need to boost ego, social pressure, and rapid reward processes, are discussed. The implications and applications of the research are also discussed, focusing on limit setting and policy development focusing on frequency of micro-transaction use.

## Introduction

In recent years, there has been concern for micro-transaction users in relation to problematic gambling and gaming behaviours. This has particularly concerned young people and the ease of access to micro-transactions in videogames. A recent report published by the Gambling Commission (2022) highlighted that 39% of 11–16-year-olds (*n* = 2299) were aware of, and had used, in-game items including skins and loot boxes. This presents concern due to associations between loot box use and problem gambling (Griffiths, 2018; Wardle & Zendle, 2021; Zendle & Cairns, 2018; Zendle et al., 2019) and the prevalence of gambling among youth populations (Calado et al., 2017; Turner et al., 2018; Zendle et al., 2019). Although broadly discussed, there is a dearth of literature regarding motivations for engaging in micro-transaction use. To understand the best approach for harm prevention or intervention measures, underpinning motivations for micro-transaction use must be considered because motivation is a crucial aspect of behavioural change (Flannery, 2017). Therefore, the present study aimed to provide a base for the development of motivational theory surrounding the use of micro-transactions and provide recommendations for both healthcare providers and videogame companies, when assessing the use of micro-transactions in videogames.

Micro-transactions remain one of the largest monetisation forms in videogames, with a projected global market growth from $67.94 billion to $76.66 billion in 2023 (The Business Research Company, 2023). Micro-transactions can be described as a form of in-game monetisation, usually consisting of low-cost virtual items or purchases that add extra content to a videogame (Gibson et al., 2023). Monetisation methods in videogames are ever-changing, with micro-transactions covering a wide range of content, from cosmetic items that change how an avatar looks and weapons that increase player skill to mechanisms such as loot boxes and battle passes, which allow for extra gameplay experiences. In the case of loot boxes, players typically obtain or purchase ‘keys’ to unlock boxes or crates that contain random cosmetic items, weapons, or boosts, of varying ‘rarity’ or prestige. Battle passes operate as seasonal items that allow players to unlock cosmetic items after obtaining increasing amounts of experience points (XPs).

Although there is a consensus in the literature surrounding micro-transaction use and problem gambling behaviours, links between micro-transaction use and problem gaming behaviours are less discussed (Gibson et al., 2022; Raneri et al., 2022). For example, research by King et al. (2020) suggested that youth spending on micro-transactions was not associated with problem gaming, and instead was based on peer purchasing behaviour. However, recent literature has suggested that gaming disorder symptoms among adolescent populations increase with the use of micro-transactions, including the purchase of loot boxes (Hing et al., 2023). Moreover, Lelonek-Kuleta et al. (2021) reported an association between pay-to-win micro-transactions and problem gaming, although it should be noted that their research focused on adult populations and so may not relate to youth populations.

Research concerning adult populations of micro-transaction users has focused on loot boxes and battle passes. Most notably, it has been suggested that loot boxes have commonalities with forms of gambling likes slot machines (Griffiths, 2018). Where slot machines use real-world currency, loot boxes are opened directly with in-game currency or using keys purchased with in-game currency (Uddin, 2021) and have similar reward schedules (Drummond & Sauer, 2018; Griffiths, 2018; von Meduna et al., 2020). Moreover, loot boxes share similar visual and audio cues to slot machines, potentially used to invoke feelings of excitement and to build anticipation before a prize is revealed (Whittaker, 2019). Research suggests that increased levels of arousal (identified through increased skin conductance response), feelings of reward, and urge to continue using loot boxes is impacted by rarity of rewards obtained (Larche et al., 2021). This could be argued to be similar to the impact of gambling, where ‘wins’ are shown to increase skin conductance response levels (Wilkes et al., 2010).

Research into the psychological impact of the battle pass mechanism has primarily focused on the prevalence of battle passes in games and on player perceptions of the battle pass. A mixed methods study by Petrovskaya and Zendle (2020) found that the inclusion of a battle pass mechanism in the game *Dota 2* did not significantly impact player engagement but that player spending on battle passes steadily increased. It is suggested that spending on battle passes has increased due to the ability to ‘level up’ the battle pass using experience points (XPs) or by purchasing levels. Consequently, player engagement with the game may not be impacted by the battle pass, but spending on battle passes may have increased due to the ability to unlock levels quickly and easily through purchasing, rather than players actively earning XPs to gain rewards. This was further discussed in Petrovskaya and Zendle's (2020) research, which also addressed *Dota* 2 player perceptions of the battle pass. It was found that attitudes towards battle passes were mainly negative, with discussion centring around the *spend-grind trade off* and *elitism*. Players discussed the need to spend money unlocking the levels, due to unachievable XP goals. This then led to feelings of elitism in-game, whereby players who were unable to unlock items through paying discussed feeling left out and ‘closed off’ from the ‘elite’ community members.

Similar negative attitudes to micro-transaction use were discussed by Gibson et al. (2023) when assessing videogame player motivations for micro-transaction use. Main themes included feelings of guilt when using micro-transactions, being ‘tricked’ by micro-transactions, and having a sense of obligation to play a game for longer if micro-transactions were used. Participants also described the similarities between gambling and loot box use, as well as the euphoric and rewarding feelings of engaging in a micro-transaction, leading to the potential need to purchase micro-transactions more frequently.

Frequency of micro-transaction use and potential links to problematic gaming and gambling behaviour have been discussed in literature, although there is currently a lack of consensus regarding the role frequency of micro-transaction use may play in the development of problematic behaviour. Gibson et al. (2022) discussed frequency of micro-transaction use as a potentially important factor in the development of problematic behaviour, particularly concerning reward event frequency and allowing for pauses between reward reinforcements. In the wider literature, higher frequencies of gambling events are suggested to increase levels of arousal, enjoyment and potentially contribute to impulsive behaviour through impaired motor response inhibition (Harris & Griffiths, 2018; Harris et al., 2021).

Similarly, the structural characteristics of videogames are suggested to operate using highly engaging reward systems through fast loading times (i.e., high frequencies of events), the earning of XPs, levelling up, or obtaining rewards and loot from competing videogame quests or storylines, which increases enjoyment of and engagement with gaming, leading to potentially problematic behaviour (King et al., 2011).

It has also been suggested that increased spending on micro-transactions can lead to problematic behaviour. There is consensus that there is an association between expenditure on micro-transactions (more specifically, buying loot boxes) and problem gambling behaviour, with suggestions that as expenditure on loot boxes increases, so does problem gambling severity (Drummond et al., 2020; Zendle et al., 2019). It is argued that this is due to the structural similarities between loot boxes and gambling forms such as slot machines (Raneri et al., 2022). However, there is limited research evidencing a link between expenditure on micro-transactions and problematic gaming behaviour. Research by Drummond et al. (2020) suggests that those exhibiting symptoms of *both* gaming disorder and problem gambling were more likely to spend higher amounts of money on loot boxes than those with symptoms aligning with either gaming disorder *or* problem gambling. This could indicate a more complex interaction between problematic gaming and gambling and the role that micro-transaction use has on this relationship. However, further research is necessary to explore the interaction between gaming, gambling, and micro-transaction use.

Although research surrounding micro-transaction use is becoming more prominent, the relationships between self-determined motivations, elements of micro-transaction use (i.e., spend amounts, frequency of use and micro-transaction type) and problem gaming and gambling have not yet been explored in-depth.

## Theoretical Framework and Hypotheses

### Self-Determination Theory (SDT)

SDT is a macro-theory concerned with the concept of human development and ‘active’ growth through intrinsic motivation (i.e., the tendency to seek out challenges and learning opportunities) (Deci & Ryan, 2015). SDT comprises five mini-theories of motivation and personality: causality orientations theory, goal contents theory, cognitive evaluation theory, basic psychological needs theory, and organismic integration theory (Ryan & Deci, 2000a).

SDT posits that motivations can be autonomous or controlled (Deci & Ryan, 2008). These motivations lie on a spectrum from amotivation (i.e., a lack of control) and extrinsic (i.e., dependent on external pressures and reward) to intrinsic (i.e., for an individual’s enjoyment or to build skill). That is, motivations can be more self-determined or autonomous (intrinsic) in nature, or less self-determined and controlled in nature (extrinsic and amotivation).

Basic psychological needs theory (BPNT) posits that humans need to satisfy three basic psychological needs to flourish. These are autonomy (i.e., the need for individuals to feel that they have chosen their own behaviour), relatedness (i.e., the need to form relationships and connections) and competence (i.e., the need for mastery and success). Individuals oriented towards controlled (more closely aligned to extrinsic motivations and amotivation) motivations have been associated with maladaptive behaviours that limit flourishing, as well as frustration with basic psychological needs, whereas those oriented towards autonomous motivations (more closely aligned to intrinsic motivations) are suggested to be more likely to satisfy their basic psychological needs and thrive (Oostdam et al., 2019). The present study applied BPNT to the use of micro-transactions and their relationship to motivations for gaming and gambling, problem gambling and problem gaming.

### Self-Determination Theory, Gaming Motivation, and Problem Gaming Behaviours

Ryan et al. (2006) discussed the *“motivational pull”* of videogames using SDT. Their research suggests that videogame players look to satisfy their basic psychological need for autonomy, relatedness, and competence through videogame play. Findings suggest that autonomy, relatedness, and competence independently predicted future gameplay and videogame enjoyment. Moreover, those who experienced more autonomy and competence satisfaction during gameplay were shown to feel more positive benefits from gameplay, suggesting that it may be the role of frustration with basic psychological needs that results in problematic gaming and gambling behaviour (Vuorinen et al., 2022), irrespective of time spent playing a game, if the underlying motivations are more controlled in nature. In fact, it has been suggested that a frustration with daily needs is an explanation for the association between introjected regulation (a form of extrinsic motivation) and amotivated gaming with problem videogame playing, whereby a frustration with autonomy partially mediates the relationship (Mills et al., 2018).

Moreover, it is suggested that there is a reciprocal relationship between internet gaming disorder (IGD) and need satisfaction, such that those with IGD symptoms were more likely to have lower levels of needs satisfaction over time, which in turn leads to maintenance of IGD symptoms and worsening mental and physical health (Weinstein et al., 2017). This is supported by Scerri et al.’s (2018) research, which suggested that a higher needs-fulfilment deficit (lower satisfaction with the three basic psychological needs for autonomy, relatedness, and competence) was associated with IGD behaviours, with this relationship being mediated by self-esteem levels and depression. Therefore, it could be the case that those with higher needs-fulfilment deficit utilise gaming to increase their real-world needs satisfaction, through obtaining in-game XPs, socialising, and potentially purchasing items. The process of needs fulfilment and reward with progression or items may then undermine those motivated intrinsically, or further reward those motivated extrinsically (Ryan & Deci, 2000b), leading to increased engagement and involvement in the videogame and causing the development or maintenance of IGD symptoms.

### Self-Determination Theory, Gambling Motivation, and Problem Gambling

SDT has also been discussed in relation to problematic gambling behaviour. It has been suggested that those who gamble through highly self-determined motivations are more likely to continue gambling (i.e., intrinsic motivations for gambling are associated with involvement and continuation of gambling) than those who are motivated by external reward (Chantal et al., 1995). Moreover, it has been found that skill-based gambling (i.e., betting on horseraces) is more associated with self-determined motivations, whereas luck-based gambling (i.e., slot machines) is more associated with less self-determined motivations.

Conversely, other studies have found that autonomous motivation for gambling was negatively associated with problematic behaviour, whereas those who gambled through controlled motivations (i.e., extrinsic and amotivation) were more likely to gamble more frequently, spend more money on gambling, and exhibit more severe problematic behaviour (Neighbors & Larimer, 2004). This is further evidenced by Rodriguez et al. (2015) who found that autonomous motivation was negatively associated with gambling problems, where those who were autonomously motivated did not gamble to chase losses or for escapism. On the other hand, their research found that controlled motivations were typically associated with higher levels of problem gambling.

Considering the role of needs frustration, it has been proposed that external pressures (i.e., more extrinsically positioned motivations) and a lack of control (i.e., amotivation) are related to greater frustration with basic needs, which can then lead to greater psychological distress and problematic gambling behaviour (Mills et al., 2021). In this case, needs frustration may have a mediating role. However, it has also been suggested that needs frustration may moderate or predict increased severity of gambling problems when the initial motivation to gamble is for extrinsic reward, specifically monetary gain (Hagfors et al., 2023). It should also be noted that Hagfors et al.’s (2023) research was longitudinal in nature, as opposed to Mills et al.’s (2021) research, which was cross-sectional (and therefore correlational) in nature. Consequently, longitudinal research may provide a more complete picture of the role of needs frustration. However, further research to confirm the stability of the role of needs frustration may be beneficial for understanding the complex relationships involved.

### Self-Determination Theory and Micro-Transaction Use

To establish and develop theory surrounding micro-transaction use, research has assessed motivations for micro-transaction use. Typically, with a focus on loot boxes (Nicklin et al., 2021; Zendle et al., 2019) or more recently, with battle passes (Petrovskaya & Zendle, 2020). Gibson et al.’s (2023) study reported motivations for the use of multiple forms of micro-transactions, including those that are expiration based (i.e., extra lives or time), loot boxes, single purchase (i.e., skins or cosmetic items), battle passes, and in-game currency. The most prominent motivations for purchase were rewarding developers, limited time offers, peer influence, and social status. Both Gibson et al. (2023) and Nicklin et al. (2021) associated motivations for the use of micro-transactions with self-determination theory (Ryan & Deci, 2000a).

Research regarding SDT and micro-transaction use is in its infancy. However, it has been suggested that in-game spending is driven by a frustration with the basic psychological needs, with relatedness (i.e., the need for a sense of belonging, community, and friendship) and competence (i.e., feelings of mastery or achievement) as underpinning motivations for micro-transaction use (Lemmens & Weergang, 2023).

## The Present Study (Aims and Hypotheses)

Based on the aforementioned literature and the research gaps identified, the present study had one main research objective, which was to examine the role of micro-transactions in the relationship between self-determined motivations for gaming and gambling and problematic behaviour (internet gaming disorder and problem gambling severity). It also had a number of hypotheses. In relation to the direct effects of micro-transactions, it was hypothesized that: (i) both extrinsic motivation and amotivation would predict higher frequency of micro-transaction use (H_1a_), (ii) both extrinsic motivation and amotivation would predict higher spend per micro-transaction (H_1b_), (iii) intrinsic motivation would negatively predict frequency of micro-transaction use (H_2a_), and (iv) intrinsic motivation would negatively predict spend per micro-transaction (H_2b_).

In relation to the indirect effects of micro-transactions, it was hypothesized that: (i) both extrinsic motivation and amotivation would positively predict frequency of micro-transaction use, which predicts higher severity of problem behaviour (H_3a_), (ii) both extrinsic motivation and amotivation positively would predict spend per transaction, which predicts higher severity of problem behaviour (H_3b_), (iii) intrinsic motivation would negatively predict frequency of micro-transaction use, which predicts lower severity of problem behaviour (H_4a_), (iv) intrinsic motivation would negatively predict spend per transaction, which predicts lower severity of problem behaviour (H_4b_), (v) both extrinsic motivation and amotivation would positively predict higher levels of needs frustration, which further predicts higher frequency of micro-transaction use, which is then positively associated with problem behaviour (H_5a_), (vi) both extrinsic motivation and amotivation would positively predict higher levels of needs frustration, which further predicts higher spend per transaction, which is then positively associated with problem behaviour (H_5b_), (vii) intrinsic motivation would positively predict higher levels of needs satisfaction, which further predicts lower frequency of micro-transaction use, which is then negatively associated with problem behaviour (H_6a_), and (viii) intrinsic motivation would positively predict higher levels of needs satisfaction, which further predicts lower spend per transaction, which is then negatively associated with problem behaviour (H_6b_).

In relation to the moderation effects, it was hypothesized that: (i) the type of micro-transaction used would strengthen the relationship between frustration or satisfaction with needs and frequency of micro-transaction use (H_7a_), (ii) the type of micro-transaction used would strengthen the relationship between frustration or satisfaction with needs and spend per transaction (H_7b_), (iii) the type of micro-transaction used would strengthen the relationship between micro-transaction frequency and spend and the severity of problem behaviour (H_8a_) and (iv) the type of micro-transaction used would strengthen the relationship between micro-transaction frequency and spend and the severity of problem behaviour (H_8b_).

## Method and Materials

### Design and Participants

Data were collected from January 2023 to March 2023 using an online survey hosted on the *Qualtrics* platform. Participants were recruited using social media platforms *Twitter* (now *X*) and *Reddit*. Students from the authors’ employing university were also invited to take part using a *Microsoft Teams* link.

Participants were eligible to take part if they (i) were aged 18 years old and over, (ii) currently played or have previously played videogames and (iii) currently used or have previously used micro-transactions. The eligibility criteria needed to be met for participants to take part in the study. Participants were informed that they could only take part and be entered into a prize draw for compensation if they were living in the UK. However, participants outside of the UK who wished to take part could do so voluntarily without being entered into the prize draw. The study was approved by Nottingham Trent University’s School Research Ethics Committee in December 2022.

Power analysis suggested that a sample size of 308 would provide adequate power to estimate the proposed model, with an anticipated medium effect size of 0.3 and a statistical power level of 0.8 (Soper, 2023). A total of 646 participants were initially recruited to take part in the survey. Of the 646 responses received, 18 were removed due to being perceived as non-serious responses (e.g., participants reporting their age as ’99 years’) and 258 were removed due to being flagged as ‘bot’ responses. Bot responses were detected using *Qualtrics’* built-in bot detection software, which uses reCAPTCHA technology to calculate a score between 0 and 1 for each participant, where scores below 0.5 indicate that the response is a non-valid bot response. In the present study, responses flagged with a score below 0.5 were removed from the sample. This left a final sample of 370 participants. The sample was predominantly male (*n* = 274, 74.1%) and the sample was aged between 18 and 65 years (M_age_ = 28.24 years, SD = 7.88). Further demographic information can be found in Table 1.

**Table 1:** Participant demographic information

Descriptive	Category	Frequency	Percentage (%)
Gender			
	Male	274	74.1
	Female	71	19.2
	Non-binary/third gender	20	5.4
	Prefer not to say	5	1.4
Age (in years)			
	25–34	167	45.1
	18–24	139	37.6
	35–44	48	13.0
	45–54	12	3.2
	55–64	3	0.8
	65 and older	1	0.3
Employment status			
	Employed-full time	193	52.2
	Student	83	22.4
	Employed-part time	38	10.3
	Unemployed-looking for work	24	6.5
	Unemployed-not looking for work	11	3.0
	Other	7	1.9
	Prefer not to say	9	2.4
	Retired	5	1.4
Preferred platform			
	PC only	158	42.7
	Console only	59	15.9
	PC and console	48	13.0
	PC and mobile	47	12.7
	PC, console, and mobile	24	6.5
	Mobile only	18	4.9
	Console and Mobile	16	4.3
Time spent gaming per week			
	Less than 1 h	1	0.3
	1–5 h	50	13.5
	6–10 h	77	20.8
	11–15 h	89	24.1
	16–20 h	63	17.0
	More than 20 h	88	23.8
	Unsure	2	0.5
Preferred micro-transaction type			
	Skins or other single purchase cosmetic items	119	32.2
	Multiple micro-transactions	93	25.1
	Battle passes	89	24.1
	Loot boxes	44	11.9
	Expiration-based (e.g., extra lives or level skips)	25	6.8
Frequency of micro-transaction purchase(s)			
	Daily	3	0.8
	Weekly	24	6.5
	Monthly	125	33.8
	Annually	107	28.9
	Less frequently than annually	111	30.0
Typical micro-transaction spend per transaction			
	£0	13	3.5
	< £1	12	3.2
	£1-4.99	82	22.2
	£5-9.99	119	32.2
	£10-19.99	95	25.7
	£20-£39.99	34	9.2
	£40-59.99	10	2.7
	£60-100	4	1.1
	> £100	1	0.3
Gambling type in past 12 months			
	National lottery draw	106	18.6
	Online betting	42	7.4
	Another lottery	38	6.7
	Slot machines	30	5.3
	Horse races	29	5.1
	Online gambling	25	4.4
	Bingo	24	4.2
	Other betting with a bookmaker	19	3.3
	Private betting	19	3.3
	Betting pools	18	3.2
	Casino	17	3.0
	Spread betting	4	0.7
	Dog races	3	0.5
	Fixed odds betting tables	2	0.4
	Betting exchange	1	0.2
	None of the above	192	33.7

### Measures

The online survey administered to participants comprised basic demographic questions, demographic-style videogame and gambling engagement questions, psychometric measures (assessing motivation, problem gaming, and problem gambling), and demographic-style micro-transaction use questions. All psychometric measures used in the present study had been previously developed and psychometrically validated.

### Demographic Information

Participants were asked basic demographic information, such as their age, gender, employment status, and annual income.

### Videogame and Gaming Engagement

Participants were asked to answer demographic-style questions about their videogame use, including their preferred gaming platform (i.e., PC, console or mobile device) and how many hours a week they typically spent playing videogames. Participants who responded that they had taken part in any form of gambling in the previous 12 months were asked to select which from 16 different forms (found in Table 1). If participants selected ‘none’, they were not asked any gambling-related questions.

### Gaming Motivation Scale (GAMS)

The GAMS (Lafrenière et al., 2012) is an 18-item scale assessing motivations for gaming. Scale items are rated using a seven-point Likert scale (ranging from 1 = *Do not agree at all*, to 7 = *Very strongly agree*) to assess *intrinsic motivation* (e.g., *“I play games for the feeling of efficacy I experience when I play”*), four sub-scales of extrinsic motivation, including *integrated regulation* (e.g., *“I play because it is an extension of me”*), *identified regulation* (e.g., *“I play because it is a good way to develop important aspects of myself”*), *introjected regulation* (i.e., I play because I feel that I must play regularly) and *external regulation* (e.g., *“I play for the prestige of being a good player”*) and *amotivation* (i.e., *“Honestly, I don’t know why I play”*). The GAMS is scored by summing the total score for each motivation subscale, with a maximum possible score of 21 per motivation type. Cronbach’s alpha for each subscale was calculated by Lafreniere et al. (2012) to range from 0.75 to 0.89. In the present study, Cronbach’s alpha for each subscale ranged from 0.71 to 0.90, except for intrinsic motivation which had a Cronbach’s alpha of 0.48. Consequently, during the first stage of model assessment, the intrinsic motivation subscale was removed from further testing.

### Modified Gambling Motivations Scale (MGMS)

The MGMS (Shinaprayoon et al., 2017) is a 28-item scale assessing motivations for gambling. Scale items are rated using a seven-point Likert scale (1 = *Strongly disagree,* 7 = *Strongly agree*) to assess *intellectual challenge* (e.g., *“I enjoy improving my knowledge of the game”*), *excitement* (e.g., *“It is exciting to gamble”*), *socialisation* (e.g., *“It is the best way to relax”*), *monetary gain* (e.g., *“I play for money”*), *social recognition* (e.g., *“It makes me feel important”*) and *amotivation* (e.g., *“I play for money, but I sometimes worry if I should continue playing”*). The MGMS is scored by summing the scores for each subscale, then finding their average, with possible scores for each subscale ranging from zero to seven, and a total maximum score of 42. Higher scores for each subscale indicate a higher motivation to gambling for that particular motivation type, and a higher total score indicates a higher level of motivation to gamble in general. Cronbach’s alpha for each subscale was calculated by Shinaprayoon et al. (2017) to range from 0.76 to 0.92. In the present study, Cronbach’s alpha for each subscale ranged from 0.95 to 0.98.

### Internet Gaming Disorder Scale—Short Form (IGDS-SF9)

The IGDS-SF9 (Pontes & Griffiths, 2015) is a nine-item scale assessing internet gaming disorder. Scale items are rated using a five-point Likert scale (1 = *Never*, 5 = *Very Often*) to assess each item (e.g., *“Do you feel preoccupied with your gaming behavior?”*). Scores for each item are summed, allowing for scores ranging from 9 to 45, with higher scores indicating a greater risk of IGD. Those who obtain a score of 36 and over are suggested to be classified as a disordered gamer. Cronbach’s alpha was calculated by Pontes and Griffiths (Pontes & Griffiths, 2015) to be 0.87. In the present study, Cronbach’s alpha was 0.86.

### The Problem Gambling Severity Index (PGSI)

The PGSI (Ferris & Wynne, 2001) is a nine-item scale assessing problem gambling. Scale items are rated using a four-point scale (0 = *Never*, 3 = *Almost always*) to assess each item (e.g., *“Have you bet more than you could really afford to lose?”*). Item scores are summed, ranging from a minimum total score of 0 and a maximum total score of 27. Those who score eight or more are suggested to be problem gamblers. Those who score between 3 and 7 are suggested to be moderate risk gamblers (i.e., exhibiting moderate levels of problematic behaviour and experiencing some negative consequences). Those who score 1 or 2 on the scale are suggested to be low-risk gamblers (i.e., exhibiting low levels of problematic behaviour, with limited or no negative consequences). Cronbach’s alpha was calculated by Ferris and Wynne (Ferris & Wynne, 2001) to be 0.84. In the present study, Cronbach’s alpha was 0.95.

### Basic Psychological Needs Satisfaction/Frustration Scale (BPNSFS)

The BPNSFS (Chen et al., 2015) is a 24-item scale assessing satisfaction and frustration with the basic psychological needs of autonomy, competence, and relatedness. Each of the three basic psychological needs has eight items, four that assess satisfaction and four that assess frustration. Scale items are rated using a five-point Likert scale (1 = *Completely false*, 5 = *Completely true*) to assess each item (e.g., *“I feel a sense of choice and freedom in the things I undertake”*). The score for item from each subscale is summed to produce a total score for autonomy, relatedness, and competence (for both needs satisfaction and needs frustration). Scoring for the BPNSFS can be utilised in different ways, whereby composite scores can be used to assess needs satisfaction or frustration as whole concepts. The present study used composite scores for each subscale, to create one needs satisfaction construct, and one needs frustration construct. The internal reliability for each subscale was calculated across four countries (US, China, Belgium, and Peru) by Chen et al. (2015), with Cronbach’s alpha ranging from 0.64 to 0.89. In the present study, Cronbach’s alpha for the needs frustration subscale was 0.90 and Cronbach’s alpha for the needs satisfaction subscale was 0.89.

#### Micro-Transaction Use

Micro-transaction use was assessed using three separate demographic-style questions. These were: (i) micro-transaction type typically used (i.e., loot boxes, battle passes, cosmetic items, expiration items or multiple types), (ii) typical frequency of micro-transaction use (i.e., daily, weekly, monthly, annually, or less frequently than annually), and (iii) typical amount of money spent per transaction (i.e., a continuous numerical value in GBP [£]).

#### Statistical Analysis

The present study utilised R and SmartPLS 4.0 (Ringle et al., 2022) for data handling, descriptive statistics, and model analysis. SmartPLS software was used for Partial Least Squares – Structural Equation Modelling (PLS-SEM). Before commencing with analysis of the data, several key factors were considered regarding the appropriate form of SEM to undertake.

First, PLS-SEM is a model validation method that combines principal components analysis and ordinary least squares regression and is traditionally used in cases of theory development, when there is limited literature available and previous model testing has not occurred (Hair et al., 2019). Additionally, PLS-SEM is similar to running a series of multiple regression analyses. However, these are run simultaneously and relationships between variables can be more complex (Mehmetoglu, 2012). Moreover, PLS-SEM is suggested to be robust against smaller sample sizes and models with complex structures (i.e., serial mediation and moderation interactions) and higher numbers of variables (Dash & Paul, 2021). Finally, PLS-SEM is suggested to be more appropriate for the analysis of non-normally distributed data, which can often occur in social science research (Bono et al., 2017). Consequently, the present study utilised PLS-SEM methods to analyse the proposed model due to the complexity of the model structure.

To ensure that PLS-SEM was an appropriate method of model testing, an initial analysis was carried out using R packages *lavaan* (Rosseel, 2012) and *MVN* (Korkmaz et al., 2014) to identify if the cleaned data were normally distributed. Results from the Mardia’s multivariate skewness and kurtosis tests (*p* < 0.001) indicated that the data were not normally distributed. Shapiro–Wilk’s univariate test further indicated that the variables were not normally distributed. It is suggested that that Shapiro–Wilk’s univariate test is sensitive to sample sizes > 300 and consequently, absolute skewness and kurtosis (excess) values should also be analysed for non-normality, whereby skewness values should be ≤ 2 and kurtosis (excess) values should be ≤ 4 (Mishra et al., 2019). Descriptive statistics for each variable, including absolute skewness and kurtosis (excess) values can be found in Table 2. Subsequently, PLS-SEM was deemed as an appropriate method for model evaluation.

**Table 2:** Means, standard deviations, skewness, and kurtosis (excess) values

Variable	Mean	SD	Skewness	Kurtosis (excess)
Gaming: Amotivation	8.16	4.81	0.69	− 0.55
Gaming: External Regulation	13.05	4.23	− 0.4	− 0.67
Gaming: Identified Regulation	13.43	3.6	− 0.26	0
Gaming: Integrated Regulation	12.75	4.1	− 0.32	− 0.45
Gaming: Introjected Regulation	8.35	4.32	0.68	− 0.39
Gambling: Amotivation	1.28	1.78	1.37	0.9
Gambling: Extrinsic Motivation	1.15	1.37	0.81	− 0.41
Gambling: Intrinsic Motivation	1.24	1.6	1.05	− 0.09
IGD	18.73	6.25	0.99	1.12
Needs Frustration	31.5	9.43	0.12	− 0.7
Needs Satisfaction	44.35	7.68	− 0.58	− 0.22
PGSI	1.33	3.44	3.62	15.21

IGD, Internet Gaming Disorder; PGSI, Problem Gambling Severity Index

PLS-SEM typically consists of two stages of model analysis (Hair et al., 2017). The first stage of analysis focuses on testing the measurement model (i.e., assessing that items assessing latent constructs were reliable and valid at testing the underlying concept of the variable). The second stage of analysis focuses on testing the structural model (i.e., assessing the constructs and their relationship pathways). Both measurement model and structural model analysis were conducted using SmartPLS 4.0 (Ringle et al., 2022).

## Results

### Measurement Model Testing

First, item loadings were assessed. Typically, it is suggested that items have loading values larger than 0.70 (Hair et al., 2019). However, Hulland (1999) suggests that item loadings of > 0.4 are acceptable, particularly in the case of social science research. It is suggested that items loading below 0.3 are removed (Field, 2013).

Internal consistency and reliability were tested using Cronbach’s alpha and McDonald’s omega. Cronbach’s alpha and McDonald’s omega values are suggested to be acceptable above 0.7 (McNeish, 2018). Composite reliability values are also suggested to be acceptable above 0.7 (Hair et al., 2014). Convergent validity was assessed using average variance extracted (AVE), whereby values should be > 0.5 (indicating that over 50% of the indicator variance can be explained by the latent variable). However, it is suggested that AVE is a more conservative measurement of validity, and consequently should not be used to assess convergent validity alone. Instead, composite reliability scores can also be used to establish convergent validity (Fornell & Larcker, 1981). In the case where AVE values are < 0.5, it is suggested by Hair et al. (2017) that constructs with an AVE value of > 0.4 and a composite reliability of > 0.6 are acceptable, but any items with loadings of < 0.3 should be removed. Subsequently, one item from the problematic gaming variable was removed (“*Do you play in order to temporarily escape or relieve a negative mood (e.g., helplessness, guilt, anxiety)?*”)*.* Once removed, AVE and composite reliability increased to acceptable levels.

The intrinsic gaming motivation variable was found to not be reliable (α = 0.48, ω = 0.51). Low reliability may occur when a factor has a small number of items, items do not accurately assess the underlying construct, or items assess a complex construct (Tavakol & Dennick, 2011). In the case of the intrinsic subscale of the GAMS (Lafrenière et al., 2012), it is possible that participants were not clear on the wording of the items, leading to responses that did not accurately capture the underlying concept of intrinsic gaming motivation. For example, Item 3 (“For the feeling of efficacy I experience when I play”) uses vague wording (i.e., the definition of efficacy may not be fully understood by participants), as was suggested by Shinaprayoon et al. (2017).

Item deletion was not considered due to the factor consisting of only three items, which is suggested to be the minimum number of items for a stable factor (Raubenheimer, 2004). Furthermore, it is suggested that factors are only retained when they contain more than three items and can be meaningfully interpreted (Worthington & Whittaker, 2006). Wider gaming research using the intrinsic motivation subscale of the GAMS (Lafrenière et al., 2012) has also encountered issues with the reliability of the subscale, resulting in its exclusion (Mills et al., 2018). Consequently, the intrinsic gaming motivation variable was excluded from the model. Final Cronbach’s alpha, McDonald’s omega, compositive reliability and AVE values can be found in Table 3.

**Table 3:** Cronbach's alpha, composite reliability, and AVE values

Variable	Cronbach's alpha	McDonald’s Omega	Composite reliability	Average variance extracted (AVE)
Gaming: Amotivation	0.90	0.90	0.90	0.83
Gaming: External Regulation	0.71	0.71	0.70	0.63
Gaming: Identified Regulation	0.71	0.73	0.70	0.63
Gaming: Integrated Regulation	0.80	0.81	0.80	0.71
Gaming: Introjected Regulation	0.83	0.83	0.80	0.74
Gambling: Amotivation	0.95	0.95	1.00	0.88
Gambling: Extrinsic Motivation	0.97	0.96	1.00	0.77
Gambling: Intrinsic Motivation	0.98	0.98	1.00	0.83
IGD	0.86	0.86	0.90	0.51
Needs Frustration	0.90	0.90	0.90	0.47
Needs Satisfaction	0.89	0.88	0.90	0.44
PGSI	0.95	0.94	0.90	0.70

IGD, Internet Gaming Disorder; PGSI, Problem Gambling Severity Index

When assessing discriminant validity of the variables, it is suggested that items should be below the heterotrait-monotrait ratio of correlations of < 0.90 when variables are conceptually similar (Henseler et al., 2015). The HTMT values can be found in Table 4. In the case that some constructs are identified as too similar, item cross-loadings should be assessed during the analysis process. In the case where item cross-loadings exceed 0.90 or items present assessed another construct with less than 0.10 difference between the cross-loading construct and the parent construct, it is suggested that items should be removed (Ximénez et al., 2022).

**Table 4:** HTMT ratio values

Variable	Freq	G.Amo	G.Exter	G.Iden	G.Integ	G.Intro	Gb.Amo	Gb.Extrinsic	Gb.Intrinsic	IGD	MMT	NF	NS	PGSI
Freq														
G.Amo	0.145													
G.Exter	0.302	0.247												
G.Iden	0.079	0.174	0.246											
G.Integ	0.115	0.113	0.250	0.863										
G.Intro	0.304	0.494	0.454	0.352	0.503									
Gb.Amo	0.196	0.221	0.221	0.036	0.138	0.178								
Gb.Extrinsic	0.263	0.258	0.221	0.075	0.163	0.316	0.880							
Gb.Intrinsic	0.188	0.154	0.217	0.044	0.130	0.184	0.837	0.906						
IGD	0.315	0.553	0.328	0.152	0.320	0.679	0.203	0.279	0.181					
MMT	0.072	0.015	0.036	0.042	0.113	0.077	0.080	0.034	0.013	0.041				
NF	0.115	0.396	0.186	0.141	0.174	0.446	0.140	0.127	0.086	0.536	0.058			
NS	0.093	0.277	0.133	0.165	0.106	0.301	0.082	0.066	0.060	0.424	0.068	0.757		
PGSI	0.329	0.391	0.157	0.061	0.122	0.379	0.590	0.671	0.499	0.462	0.031	0.226	0.191	
Spend	0.122	0.120	0.092	0.047	0.047	0.083	0.078	0.093	0.117	0.146	0.023	0.090	0.104	0.034

HTMT, Heterotrait-Monotrait; Freq, Frequency; G.Amo, gaming amotivation; G. Exter, gaming external regulation; G.Iden, gaming identified regulation; G.Integ, gaming integrated regulation; G.Intro, gaming introjected regulation; Gb.Amo, gambling amotivation; Gb.Extrinsic, extrinsic gambling motivation; Gb.Intrinsic, intrinsic gambling motivation; IGD, internet gaming disorder; MMT, micro-transaction type; NF, needs frustration; NS, needs satisfaction; PGSI, Problem Gambling Severity Index; Spend, micro-transaction spend

Consequently, three items were removed from the intrinsic gambling motivation variable due to high cross loading (i.e., “*I feel competent when I gamble*”, “*Gambling allows me to test my control*” and “*It gives me a feeling of control*”). A further two items were also removed from the extrinsic gambling motivation variable (i.e., “*I play for money*” and “*I feel important when I win*”). Once these were removed, variables were equal to or below the suggested 0.90 threshold. For any variables with a HTMT ratio equalling 0.90, any cross-loading between items had a difference of more than 0.10 between the cross-loading variable and the parent variable and consequently, were deemed acceptable. When assessing why items may cross-load, it may be beneficial to consider the theoretical underpinnings of the variables in the present study. That is, self-determined motivations. These are suggested to lie on a spectrum from completely extrinsic to completely intrinsic, with some forms of motivations linked to both extrinsic and intrinsic (i.e., the integrated regulation style of motivation). Consequently, it may be the case that items cross-load when they are theoretically linked, as was the case with present study. However, to avoid issues with discriminant validity, items were removed in the present study. Once the convergent reliability and validity and discriminant validity levels were acceptable, the next stage of the PLS-SEM analysis were conducted.

### Structural Model Testing

According to Hair et al. (2014), best practice for SEM requires testing the structural model for common method bias by assessing if there is multicollinearity between independent variables. This is carried out by assessing the variance inflation factor (VIF) of endogenous variables. Suggested values for VIF are below 5 or below 10 (James et al., 2013). However, there is little consensus regarding adequate values, and this varies by discipline. All VIF values were under 10, indicating minimal issues with collinearity.

Model predictive power and in-sample explanatory power are then assessed using R^2^ and Q^2^ values. R^2^ values must be above 0.10 for variance of the endogenous construct to be explained (Falk & Miller, 1992). The adjusted R^2^ values indicate that the variance for all endogenous variables, other than spend (R = 0.010), were an adequate value (Table 5).

**Table 5:** Adjusted R^2^ and Q^2^ values for each endogenous variable

Variable	Adjusted R^2^	Q^2^
Frequency	0.136	0.084
IGD	0.475	0.373
Needs Frustration	0.195	0.160
Needs Satisfaction	0.144	0.105
PGSI	0.524	0.455
Spend	0.010	− 0.043

IGD, Internet Gaming Disorder; PGSI, Problem Gambling Severity Index

It is suggested that Q^2^ values are indicative of predictive power of the endogenous constructs and the model as a whole (Henseler et al., 2009). Q^2^ values above 0 typically indicate good predictive power of the constructs. It should be noted that the Q^2^ value for the spend construct was below 0 and consequently, was not useful for in-sample prediction. However, all other values were adequate (Table 5).

Finally, pathway analysis was performed using two-tailed bootstrapping (with 5000 resamples) with a confidence interval of 95%. The following section outlines the results of the pathway analysis and subsequently, the hypothesis testing stage of analysis.

### Hypothesis Testing

This section outlines the key findings from the hypothesis testing process. Several significant relationships were found, although no serial mediation or moderation interactions were observed at a significant level. Instead, direct, and simple mediation effects were found to be statistically significant.

### Statistically Significant Findings

Of the hypotheses tested, two were partially supported. These related to the relationship between motivations for gaming and gambling, frequency of micro-transaction use, and gambling behaviour.

The first partially supported hypothesis (H_1_) was that both extrinsic motivation and amotivation would predict higher frequency of micro-transaction use. In the present study, this was the case for externally regulated extrinsic gaming motivation (β = 0.2, *p* < 0.01), introjected regulation gaming motivation (β = 0.147, *p* = 0.046), and extrinsic gambling motivation (β = 0.311, *p* = 0.007). The remaining forms of extrinsic gaming motivation (i.e., identified regulation and integrated regulation), as well as amotivation did not significantly predict higher frequencies of micro-transaction use.

The second partially supported hypothesis (H_5a_) was that frequency of micro-transaction use would mediate the relationship between both extrinsic motivation and amotivation for gaming and gambling and problematic gaming and gambling behaviour. In the present study, frequency of micro-transaction use partially mediated the relationship between extrinsic motivations for gambling and problem gambling severity (β = 0.05, *p* = 0.032).

Finally, a further significant finding emerged that was not initially hypothesised. That is, the relationship between extrinsic gaming motivations and problem gambling. It was found that frequency of micro-transaction use fully mediated the relationship between external gaming motivations and problem gambling severity (β = 0.032, *p* = 0.01).

### Statistically Non-significant Findings

None of the hypotheses related to micro-transaction expenditure were found to be significant during the pathway analysis stage of the present study. Moreover, no serial mediation pathways were found to be significant (i.e., needs frustration and satisfaction did not play a role in the relationship between self-determined motivations, frequency of micro-transaction use, and problematic gaming and gambling behaviour).

Similarly, the use of multiple forms of micro-transaction was not found to have a statistically significant moderating effect on needs frustration and satisfaction, frequency of micro-transaction use, and micro-transaction expenditure pathways. Table 6 contains further information about all pathways analysed and their statistical significance. It should be noted that the intrinsic gaming motivation variable was excluded during the data analysis stage, and consequently, pathways relating to intrinsic gaming motivation are not presented and discussed.

**Table 6:** Pathways analysis and hypothesis testing

Hypothesis	Pathway(s)	Std Beta (β)	Standard deviation (STDEV)	*t-*value	*p-*value	BC LL	BC UL	Hypothesis Statement
Direct effects								
H_1a_								Partially Supported
	Gb.Extrinsic—> Freq	0.311	0.116	2.686	0.007	0.081	0.539	Supported
	G.Exter—> Freq	0.2	0.054	3.676	< 0.001	0.089	0.3	Supported
	G.Intro—> Freq	0.147	0.074	1.999	0.046	0.007	0.297	Supported
	G.Integ—> Freq	− 0.001	0.066	0.019	0.985	− 0.131	0.126	Not supported
	G.Iden—> Freq	− 0.017	0.07	0.245	0.806	− 0.164	0.11	Not supported
	Gb.Amo—> Freq	− 0.037	0.09	0.416	0.678	− 0.212	0.141	Not supported
	G.Amo—> Freq	− 0.037	0.062	0.593	0.553	− 0.156	0.084	Not supported
H_1b_								Not Supported
	G.Amo—> Spend	0.117	0.078	1.487	0.137	− 0.027	0.275	Not supported
	G.Integ—> Spend	0.091	0.078	1.171	0.242	− 0.068	0.233	Not supported
	Gb.Amo—> Spend	0.037	0.089	0.416	0.677	− 0.13	0.224	Not supported
	G.Exter—> Spend	0.016	0.076	0.207	0.836	− 0.15	0.144	Not supported
	G.Intro—> Spend	0.015	0.083	0.177	0.859	− 0.15	0.172	Not supported
	G.Iden—> Spend	− 0.028	0.082	0.338	0.735	− 0.199	0.125	Not supported
	Gb.Extrinsic—> Spend	− 0.093	0.083	1.128	0.259	− 0.261	0.062	Not supported
H_2a_								Not Supported
	Gb.Intrinsic—> Freq	− 0.115	0.104	1.107	0.268	− 0.316	0.093	Not supported
H_2b_								Not Supported
	Gb.Intrinsic—> Spend	− 0.095	0.075	1.26	0.208	− 0.237	0.063	Not supported
Indirect effects								
H_3a_								Partially Supported
	Gb.Extrinsic—> Freq—> PGSI	0.05	0.023	2.148	0.032	0.013	0.105	Supported
	G.Exter—> Freq—> IGD	0.022	0.012	1.945	0.052	0.005	0.052	Not supported
	G.Intro—> Freq—> IGD	0.017	0.011	1.492	0.136	0.001	0.049	Not supported
	G.Integ—> Freq—> IGD	0	0.008	0.018	0.985	− 0.017	0.015	Not supported
	G.Iden—> Freq—> IGD	− 0.002	0.008	0.235	0.814	− 0.023	0.011	Not supported
	G.Amo—> Freq—> IGD	− 0.004	0.007	0.555	0.579	− 0.023	0.008	Not supported
	Gb.Amo—> Freq—> PGSI	− 0.006	0.015	0.406	0.685	− 0.038	0.022	Not supported
H_3b_								Not Supported
	G.Amo—> Spend—> IGD	0.006	0.008	0.723	0.47	− 0.001	0.035	Not supported
	G.Integ—> Spend—> IGD	0.005	0.007	0.659	0.51	− 0.002	0.031	Not supported
	G.Intro—> Spend—> IGD	0.001	0.006	0.136	0.892	− 0.007	0.012	Not supported
	G.Exter—> Spend—> IGD	0.001	0.005	0.156	0.876	− 0.006	0.016	Not supported
	Gb.Extrinsic—> Spend—> PGSI	0	0.003	0.154	0.877	− 0.004	0.01	Not supported
	Gb.Amo—> Spend—> PGSI	0	0.003	0.072	0.942	− 0.008	0.003	Not supported
	G.Iden—> Spend—> IGD	− 0.001	0.006	0.244	0.807	− 0.023	0.005	Not supported
H_4a_								Not Supported
	Gb.Intrinsic—> Freq—> PGSI	− 0.019	0.018	1.054	0.292	− 0.059	0.011	Not supported
H_4b_								Not Supported
	Gb.Intrinsic—> Spend—> PGSI	0	0.003	0.143	0.886	− 0.004	0.011	Not supported
H_5a_								Not Supported
	Gb.Amo—> NF—> Freq—> PGSI	0.002	0.004	0.549	0.583	− 0.003	0.011	Not supported
	G.Amo—> NF—> Freq—> IGD	0.002	0.003	0.551	0.581	− 0.002	0.01	Not supported
	G.Intro—> NF—> Freq—> IGD	0.002	0.003	0.57	0.569	− 0.003	0.011	Not supported
	G.Integ—> NF—> Freq—> IGD	0	0.001	0.135	0.892	− 0.001	0.004	Not supported
	G.Exter—> NF—> Freq—> IGD	0	0.001	0.309	0.757	− 0.004	0	Not supported
	G.Iden—> NF—> Freq—> IGD	0	0.001	0.317	0.751	0	0.005	Not supported
	Gb.Extrinsic—> NF—> Freq—> PGSI	− 0.002	0.003	0.498	0.618	− 0.013	0.002	Not supported
H_5b_								Not Supported
	Gb.Extrinsic—> NF—> Spend—> PGSI	0	0	0.038	0.97	− 0.001	0.002	Not supported
	G.Integ—> NF—> Spend—> IGD	0	0	0.042	0.966	0	0.002	Not supported
	Gb.Amo—> NF—> Spend—> PGSI	0	0	0.043	0.966	− 0.001	0.001	Not supported
	G.Amo—> NF—> Spend—> IGD	0	0.001	0.047	0.963	− 0.001	0.001	Not supported
	G.Exter—> NF—> Spend—> IGD	0	0	0.099	0.922	− 0.002	0	Not supported
	G.Iden—> NF—> Spend—> IGD	0	0	0.112	0.911	0	0.002	Not supported
	G.Intro—> NF—> Spend—> IGD	0	0.002	0.181	0.856	− 0.001	0.006	Not supported
H_6a_								Not Supported
	Gb.Intrinsic—> NS—> Freq—> PGSI	0.001	0.003	0.256	0.798	− 0.003	0.01	Not supported
H_6b_								Not Supported
	Gb.Intrinsic—> NS—> Spend—> PGSI	0	0.001	0.028	0.978	− 0.001	0.002	Not supported
Moderation								
H_7a_								Not Supported
	MMT x NS—> Freq	− 0.017	0.173	0.099	0.921	− 0.349	0.32	Not supported
	MMT x NF—> Freq	− 0.049	0.168	0.291	0.771	− 0.374	0.292	Not supported
H_7b_								Not Supported
	MMT x NS—> Spend	− 0.031	0.129	0.243	0.808	− 0.282	0.221	Not supported
	MMT x NF—> Spend	0.064	0.138	0.46	0.645	− 0.196	0.349	Not supported
H_8a_								Not Supported
	MMT x Freq—> IGD	0.024	0.11	0.217	0.828	− 0.189	0.238	Not supported
	MMT x Freq—> PGSI	− 0.124	0.077	1.618	0.106	− 0.262	0.042	Not supported
H_8b_								Not Supported
	MMT x Spend—> PGSI	0.125	0.165	0.757	0.449	− 0.045	0.587	Not supported
	MMT x Spend—> IGD	− 0.029	0.125	0.235	0.814	− 0.194	0.254	Not supported
Not hypothesised								
	G.Exter—> Freq—> PGSI	0.032	0.013	2.577	0.01	0.013	0.063	–

Freq, frequency; G.Amo, gaming amotivation; G. Exter, gaming external regulation; G.Iden, gaming identified regulation; G.Integ, gaming integrated regulation; G.Intro, gaming introjected regulation; Gb.Amo, gambling amotivation; Gb.Extrinsic, extrinsic gambling motivation; Gb.Intrinsic, intrinsic gambling motivation; IGD, internet gaming disorder; MMT, micro-transaction type; NF, needs frustration, NS, needs satisfaction; PGSI, Problem Gambling Severity Index; Spend, micro-transaction spend

## Discussion

The present study aimed to assess micro-transaction variables suggested to be involved in the relationship between self-determined motivations for gaming and gambling, and problematic gaming and gambling behaviours. Multiple key findings emerged from the present study, particularly in relation to frequency of micro-transaction use. Consequently, the following section discusses the theoretical underpinnings of the significant findings of the study. Due to the complexity of the model, the following discussion mirrors the results section of the present study. Firstly, an overview of key significant and non-significant findings is given. Potential explanations and implications of these findings are then discussed.

### Overview of Key Significant and Non-significant Findings

The pathways analysis and hypothesis testing stages of the present study highlighted three main significant findings. The first was that those motivated to play games through external and introjected regulation were more likely to engage in micro-transaction use at higher frequencies. This was also the case for those who were extrinsically motivated to gamble. The second significant finding was that frequency of micro-transaction use partially mediated the relationship between extrinsic gambling motivations and problem gambling severity. That is, frequency of micro-transaction use accounted for some (but not all) of the relationship between extrinsic gambling motivation and problem gambling severity. The final significant finding was not initially hypothesised but emerged from the pathways analysis stage of the present study. It was found that frequency of micro-transaction use mediated the relationship between externally regulated gaming motivations and problem gambling severity (i.e., frequency of micro-transaction use explained the relationship between externally regulated gaming motivations and problem gambling).

Although initially hypothesised, the role of micro-transaction expenditure was not found to be significant in the present study. Similarly, both needs frustration and satisfaction did not play significant roles in the relationships between self-determined motivations, frequency of micro-transaction use, and problematic behaviour. Finally, the use of multiple micro-transaction types was found to have no statistically significant effect on relationships between needs frustration and satisfaction, frequency of micro-transaction use, expenditure on micro-transactions, and problematic gaming and gambling behaviour.

### Significant Findings: The Relationship Between Self-Determined Motivations, Frequency of Micro-transaction Use, And Problem Behaviours

When looking at the association between extrinsic motivation and frequency of micro-transaction use, those motivated to play videogames through external regulation may be more likely to engage in micro-transaction use more frequently due to the need for external reward. In videogames, external reward may come from gaining levels or items, or gaming ‘prestige’. In this case, purchasing items and obtaining a virtual item or unlocking a level with money rather than through playing the videogame may allow the player to feel a sense of reward, without having to ‘grind’ or play the game for longer periods of time for rewards. Consequently, micro-transactions are a quicker way of achieving in-game rewards. Similarly, in the wider gambling literature, external reward is associated with the motivation to gamble for monetary gain, or to become ‘rich’, with those who gamble for financial reasons being more likely to gamble at higher frequencies (Tabri et al., 2022).

Additionally, introjected regulation-based gaming motivations focus upon self-esteem and ego (Uzun & Aydemir, 2020). For example, individuals feeling the need to play videogames regularly to feel good about themselves. In the present study, micro-transactions may enable players to earn in-game prestige and status through the rarity of rewards obtained through loot boxes or skin purchases. Introjected regulation is also suggested to be associated with obligation to perform an activity, or the feeling of needing to take part in an activity, irrespective of whether the individual wishes to (Guay, 2022). Therefore, it may be the case that participants feel like they should engage in micro-transaction use. A potential explanation for this could be that social pressure impacts micro-transaction use habits by way of peer engagement and players’ needs to ‘fit it’ with peers. In fact, King et al. (2020) found that those who experienced issues with self-worth were more likely to play videogames for longer and be influenced by peer purchasing behaviour. Moreover, internal punishments, such as feelings of guilt and anxiety are also suggested to be key factors in introjected regulation (Hurst et al., 2017). In related literature, feelings of obligation, guilt, and shame were found to be key themes related to the use of micro-transactions (Gibson et al., 2023).

It should be noted that the present study utilised gaming and gambling motivation measures, rather than motivations for micro-transaction use. At the time of writing, no such validated measure exists, and therefore, gaming and gambling measures were used, due to the nature of micro-transactions being a convergence between gaming and gambling (King & Delfabbro, 2020). It could be the case that because micro-transactions are embedded into videogames, this lends to similar motivations for micro-transaction use and gaming and gambling motivations. Suggested similarities between some gambling forms and micro-transactions may also indicate similar motivational drivers. Conversely, micro-transaction use may be its own phenomenon, but that the theoretical underpinning of SDT allows for similar motivational drivers. Further research would be beneficial to assess if those who engage in micro-transaction use have the same motivational profile as their gaming and gambling motivations.

Frequency of micro-transaction use also partially mediated the relationship between extrinsic motivations for gambling and problem gambling severity, meaning that those who use were motivated by external gambling rewards (i.e., monetary gain) were more likely to experience problems with gambling. This can partially be explained by higher frequency of micro-transaction use. It could be that the similarities between micro-transactions such as loot boxes and forms of gambling mirror the relationship between gambling ‘speed of play’ and problematic gambling behaviour. That is, the ability to rapidly engage in micro-transaction use and experience reward processes could potentially mimic highly arousing slot machine rewards schedules, whereby rewards are possible every few seconds (Griffiths & Wood, 2001). In fact, the speed of gambling play has been highlighted as a key factor in the development or maintenance of problematic gambling behaviour, particularly in the case of slot machines, whereby faster ‘rounds’ are experienced (Harris & Griffiths, 2018; Harris et al., 2021). Moreover, those who exhibit problematic behaviour typically experience higher levels of reward and loss sensitivity (Gaher et al., 2015) with those who are externally motivated being more likely to chase a loss (Lister et al., 2016).

Frequency of micro-transaction use also fully mediated the relationship between extrinsic motivations for gaming and problem gambling behaviours. That is, the relationship between those who play videogames for external reward (i.e., earning ranks, gaining rare items) and problem gambling severity can be explained by how often players engage in micro-transaction use. This could potentially indicate a causal relationship between micro-transaction use and problem gambling whereby extrinsically motivated videogame players who engage in financial micro-transactions more frequently are more likely to develop problematic gambling behaviours, irrespective of whether they engage in any form of gambling externally to the game. Conversely, and as discussed in literature, it could be the case that problem gamblers are more likely to be motivated by external rewards due to their prior involvement in gambling, and consequently, are motivated to play videogames and engage in micro-transaction use for virtual gaming rewards (Spicer et al., 2022).

### Non-significant Findings: Explanations and Implications

Firstly, it should be noted that the spend variable was not a key variable in the model tested in the present study. In fact, the variable had limited predictive and in-sample explanatory power, as well as having a Q^2^ value of below zero, indicating that the spend variable was not a significant contributor to the model. One explanation for this finding may be that spend per transaction may be relative to disposable income amounts, and not necessarily concerned with problematic behaviour. Spend amounts are suggested to be concentrated among a small number of videogame players, often titled ‘whales’ (Close et al., 2021; Hodge et al., 2022). This is similar in gambling-related literature, where it is suggested that problem gambler spend amounts are disproportionate to non-problem gamblers (Orford et al., 2013).

Needs frustration and satisfaction were not significant variables in the serial mediation pathway. That is, they did not form part of an explanation for the relationship between frequency and motivations and problematic behaviour. There are a few potential explanations for this non-significant finding. Firstly, it could be that needs frustration is addressed in real-world contexts or that micro-transactions are not used as a compensatory mechanism or need substitute (Ryan et al., 2015) to cope with a frustration with needs, as originally hypothesised. Consequently, there was no mediation effect, whereby a frustration with needs accounts for higher frequencies of micro-transaction use. T’ng et al. (2023) suggested that online gaming itself is used to provide relief from a frustration with needs, and that specific motivations for gaming mediate the relationship between needs frustration and IGD, as opposed to needs frustration as a mediator as hypothesised in the present study. Similarly, it may also be the case that needs frustration and frequency of micro-transaction use act as parallel mediators in the relationship between self-determined motivations and problematic behaviour, rather than sequential. However, further research to test this proposed model is necessary.

Alternatively, research from Hagfors et al. (2023) suggests that needs frustration has a moderating role in the relationship between gambling motivations and problem gambling. It could therefore be the case that needs frustration moderates, rather than mediates, the relationship between self-determined motivations, frequency of micro-transaction use, and problematic behaviour, such that those who engage in financial micro-transaction use more frequently and have thwarted needs, experience higher severity of problem gambling. Additionally, this could also suggest that those who are motivated extrinsically to play videogames or gamble may be more likely to engage in micro-transaction use in higher frequencies when experiencing thwarted needs. That is, needs frustration may moderate the relationship between extrinsic motivation and frequency of micro-transaction use.

Finally, the present study hypothesised that those who engaged in multiple forms of micro-transaction use, as opposed to preferring a single type, would exhibit greater problematic behaviour, as well as engage in micro-transaction use more frequently, and spend higher amounts of money on micro-transactions. However, the use of multiple micro-transaction types did not have a moderating effect on the relationship between needs satisfaction and frustration, frequency of use, and micro-transaction expenditure. Moreover, it was found that using multiple micro-transaction types had no moderating effect on the relationships between frequency of micro-transaction use and micro-transaction expenditure and problematic behaviour. In the wider gambling literature, it has been suggested that there is an association between higher gambling involvement (defined as the use of multiple forms of gambling) and problem gambling severity (Binde et al., 2017). However, it is suggested that those who only engage in one or two forms of gambling can still exhibit problem gambling behaviour, specifically in the case of those who engage with casino games (Mazar et al., 2020). Therefore, it may be the case that specific types of micro-transactions, each with their own in-game purpose and mechanisms, have different relationships to self-determined motivations and problematic behaviour (i.e., loot box use strengthens the relationship between extrinsic motivation and problematic behaviour whereas single-purchase cosmetic items have a smaller or no effect on the relationship). To test this theory, multigroup analysis could be conducted to determine the impact of different types of micro-transaction. However, this was beyond the scope of the present study.

### Limitations

The main limitation of the present study lies with the methodology used. For example, the small sample size limited the ability to carry out multigroup analysis and consequently, further research should be conducted analysing models or relationships for each type of microtransaction to identify if there are specific risks of harm from specific forms of micro-transaction. Additionally, the use of self-report data can lead to biases relating to the accuracy of responses. It may be the case that participants respond in ways they perceive to be more socially acceptable (i.e., reporting that they play games less than they do) or do not view their own behaviour accurately. Moreover, non-gamblers were more prevalent than gamblers in the sample, so this may explain lack of significant results relating to problem gambling (i.e., mediating roles of needs satisfaction and frustration in relation to problem gambling). The present study was also cross-sectional in nature and was unable to discuss longitudinal impact of motivation profile on problematic behaviour.

Additionally, PLS-SEM does not allow for the inclusion of circular or reciprocal relationships, and consequently, further research utilising CB-SEM methods could identify any reciprocal relationships between variables. For example, there may be a reciprocal relationship between frequency of micro-transaction use and problem gaming and/or gambling. Moreover, PLS-SEM does not allow for goodness of fit tests, so the present study cannot be used to confirm and test theory, only to develop and explore it. Further research could replicate the present study or develop the present study by using CB-SEM to test developed theory.

Finally, the present study used gaming and gambling scales to assess micro-transaction use motivation. It may be the case that motivation for micro-transaction use is its own phenomenon and may have differing relationships to problem gaming and gambling. Consequently, it should be assessed as a separate occurrence. Including motivations for micro-transaction use in the model, rather than separate gaming and gambling motivations may give a more accurate picture of relationships between variables. Further research should be conducted to develop and validate a micro-transaction specific motivation instrument, which could then be used to replicate the present study.

### Implications and Applications

The present study has a variety of implications and real-world applications. For example, videogame companies should consider adopting harm prevention and reduction measures for micro-transaction purchase frequency. This could be by preventing players from purchasing micro-transactions in quick succession, or providing real-world statistics of purchase frequency and spend amounts to players. By informing players and increasing time between purchases, this may prevent the development of problematic behaviours by increasing reward reinforcement time periods. Limit setting in wider gambling research has been widely discussed, highlighting potential benefits and uses for both voluntary limit setting (Auer & Griffiths, 2013) and mandatory limit setting (Delfabbro & King, 2021), so this could be adapted for the use of videogame micro-transactions.

From the results of the present study, it may be the case that those who are motivated extrinsically experience underlying issues surrounding cognitive beliefs and seek more tangible rewards, or social recognition, leading to increased engagement with micro-transactions. These underlying thought processes could be examined further and addressed through the encouragement of more intrinsically motivated activities. In this sense, interventions may benefit from focusing on behavioural changes whereby participants reflect on their own thoughts and actions, such as cognitive behavioural therapy. This may be particularly applicable for youth who exhibit problematic behaviour, allowing for the development of conscious decision-making and positive coping strategies. Focuses on well-being and increasing self-esteem may be crucial for altering motivational profiles of videogame players and ensuring harm prevention when engaging with videogames. Promotion and awareness of mindfulness from game companies may be beneficial, akin to gambling advertisements. Moreover, self-help resources could be made readily available in online gaming spaces.

Finally, policy surrounding micro-transactions should focus on limiting or regulating frequency of micro-transaction use, rather than amounts spent on in-game items. Current discourse surrounding the use of micro-transactions in games, most notably loot boxes, have focused on expenditure in videogames, rather than the frequency that purchases are made. The present study provides evidence towards frequency of use as a key factor in problematic behaviours and consequently, should be the focus of harm reduction and prevention measures.

### Areas for Future Research

Future research surrounding self-determination, motivations, and micro-transaction use may be beneficial, particularly in relation to confirmation and development of the findings of the present study. That is, the importance of frequency of micro-transaction use in the development of problematic gaming and gambling behaviour. In the present study, frustration with psychological needs was not a contributing factor in the relationship between motivations, frequency of micro-transaction use, and problematic behaviour. However, previous literature highlights that needs frustration may play a different role than hypothesised in the present study. It could be the case that other variables not specified in the present model may contribute to or exacerbate problematic behaviour. Similarly, the use of different structural equation methodology could identify any reciprocal relationships between variables, which was out of the scope of the present study.

Additionally, the present study highlighted a key gap in literature surrounding motivation for micro-transaction use, and the relationship between self-determined motivations and problem videogame playing and gambling behaviour. That is, the need for a validated motivation for micro-transaction use scale. Although micro-transaction focused scales, such as the Risky Loot Box Index (Brooks & Clark, 2019) and the Reasons and Facilitators for Loot Box Engagement Scale (Lloyd et al., 2021) are available, these focus solely on one kind of micro-transaction. A broader motivational measure would allow for wider use in a variety of healthcare and videogame user research settings.

## Conclusion

The present study utilised complex PLS-SEM methods to analyse the relationship between self-determined motivations for gaming and gambling, needs frustration and satisfaction, micro-transaction use and expenditure, and problematic behaviour. The findings indicated that frequency of micro-transaction use is a key factor in the relationship between motivations for gaming and gambling and problematic behaviour. Namely, in the case of extrinsic motivations. The present study discussed the theoretical underpinnings of the findings, including potential explanations for relationships such as a lack of self-esteem and ego, as well as the ability to rapidly engage in micro-transaction use and links to gambling. The present study also highlighted key implications and applications for the findings of the study. Most importantly, the regulation of micro-transactions and the potential need for limit setting in videogames. Policymakers and videogame companies may find the present study particularly useful for harm prevention consideration for future videogames.

## Data Availability

Data are available from the corresponding author upon reasonable request.
